# *Drosophila* Kismet Regulates Histone H3 Lysine 27 Methylation and Early Elongation by RNA Polymerase II

**DOI:** 10.1371/journal.pgen.1000217

**Published:** 2008-10-10

**Authors:** Shrividhya Srinivasan, Kristel M. Dorighi, John W. Tamkun

**Affiliations:** Department of Molecular, Cell, and Developmental Biology, University of California Santa Cruz, Santa Cruz, California, United States of America; European Molecular Biology Laboratory, Germany

## Abstract

Polycomb and trithorax group proteins regulate cellular pluripotency and differentiation by maintaining hereditable states of transcription. Many Polycomb and trithorax group proteins have been implicated in the covalent modification or remodeling of chromatin, but how they interact with each other and the general transcription machinery to regulate transcription is not well understood. The trithorax group protein Kismet-L (KIS-L) is a member of the CHD subfamily of chromatin-remodeling factors that plays a global role in transcription by RNA polymerase II (Pol II). Mutations in *CHD7*, the human counterpart of *kis*, are associated with CHARGE syndrome, a developmental disorder affecting multiple tissues and organs. To clarify how KIS-L activates gene expression and counteracts Polycomb group silencing, we characterized defects resulting from the loss of KIS-L function in *Drosophila*. These studies revealed that KIS-L acts downstream of P-TEFb recruitment to stimulate elongation by Pol II. The presence of two chromodomains in KIS-L suggested that its recruitment or function might be regulated by the methylation of histone H3 lysine 4 by the trithorax group proteins ASH1 and TRX. Although we observed significant overlap between the distributions of KIS-L, ASH1, and TRX on polytene chromosomes, KIS-L did not bind methylated histone tails in vitro, and loss of TRX or ASH1 function did not alter the association of KIS-L with chromatin. By contrast, loss of *kis* function led to a dramatic reduction in the levels of TRX and ASH1 associated with chromatin and was accompanied by increased histone H3 lysine 27 methylation—a modification required for Polycomb group repression. A similar increase in H3 lysine 27 methylation was observed in *ash1* and *trx* mutant larvae. Our findings suggest that KIS-L promotes early elongation and counteracts Polycomb group repression by recruiting the ASH1 and TRX histone methyltransferases to chromatin.

## Author Summary

The transcription of eukaryotic genes involves a highly ordered series of events, including the recruitment of RNA polymerase to promoters, the production of the RNA transcript, and termination. These events are coordinated with changes in chromatin structure that allow regulatory proteins and RNA polymerase to access the DNA template. The recruitment of RNA polymerase II to promoters is rate-limiting for the expression of most eukaryotic genes. However, RNA polymerase often pauses or stalls a short distance downstream of promoters, providing an additional step at which transcription can be regulated. In this study, we present evidence suggesting that a chromatin-remodeling factor, KIS-L, activates transcription by counteracting promoter-proximal pausing in *Drosophila*. KIS-L also counteracts histone H3 lysine 27 methylation—a covalent modification of chromatin involved in hereditable gene silencing. Our findings provide a plausible explanation for the developmental abnormalities associated with CHARGE syndrome, a serious disorder resulting from mutations in the human counterpart of KIS-L.

## Introduction

Eukaryotic transcription involves a highly ordered series of events including the binding of transcription factors to cis-regulatory elements; the assembly of the pre-initiation complex and recruitment of Pol II to promoters; initiation; promoter clearance; elongation and termination [1]. These events must be coordinated with changes in chromatin structure that allow transcription factors and Pol II to access the DNA template and the recruitment of factors that process nascent RNAs [2],[3]. The recruitment of Pol II to promoters is rate limiting for the transcription of many genes. In some cases, however, Pol II pauses or “stalls” a short distance downstream of promoters. Promoter-proximal stalling, first observed at *Drosophila* heat-shock genes, allows genes to be rapidly activated in response to cellular or environmental signals [4]. Recent studies in both *Drosophila* and humans have shown that paused polymerases are present downstream of the promoters of many silent genes, suggesting that the regulation of early elongation is a relatively widespread phenomenon [5],[6],[7],[8],[9]. In addition to poising genes for rapid induction, pausing can be used to repress transcription and generate cell type-specific patterns of gene expression [9]. These findings have stimulated great interest in the factors that regulate elongation by Pol II.

Numerous factors that regulate elongation or other aspects of transcription by Pol II have been identified in genetic studies of *Drosophila* homeotic (*Hox*) genes. *Hox* genes encode homeodomain transcription factors that specify cell fates in *Drosophila* and other metazoans [10]. The transcription of *Hox* genes must be regulated precisely, since their inappropriate expression can lead to homeotic transformations and other developmental abnormalities. The initial patterns of *Hox* transcription are established in response to positional information in the early embryo. During subsequent development, these patterns are maintained by two ubiquitously expressed groups of regulatory proteins: the trithorax group of activators and the Polycomb group of repressors [11],[12],[13]. By maintaining heritable states of *Hox* transcription, trithorax and Polycomb group proteins play key roles in the control of cell fate.

Although the detailed mechanism of action of most Polycomb and trithorax group proteins is not well understood, many of these proteins regulate transcription by altering chromatin structure. Roughly a dozen Polycomb group genes have been identified in *Drosophila*; the majority of these genes encode subunits of two protein complexes: PRC1 and PRC2 [14]. The Enhancer of zeste [E(z)] subunit of PRC2 methylates lysine 27 of histone H3 (H3K27) [15]; this covalent modification of chromatin is critical for Polycomb group repression and may recruit or stabilize the binding of PRC1 or other repressors to chromatin [16],[17]. The trithorax proteins Trithorax (TRX) and Absent, small or homeotic 1 (ASH1) also catalyze modifications of nucleosomal histones, including the methylation of lysine 4 of histone H3 (H3K4) [18],[19],[20],[21],[22]. H3K4 methylation is observed near the promoters of many active genes [23] and may directly or indirectly counteract Polycomb group repression [24],[25]. Other trithorax group proteins - including Brahma (BRM), Moira (MOR), Osa (OSA), and Kismet (KIS) - are involved in ATP-dependent chromatin remodeling [26]. Thus, both the modification and remodeling of chromatin are critical for the maintenance of heritable states of *Hox* transcription.

A subset of trithorax group members, including *kis*, may regulate elongation by Pol II. Like many other trithorax group genes, *kis* was identified in a screen for extragenic suppressors of *Polycomb* (*Pc*) mutations, suggesting that it directly or indirectly counteracts Polycomb group repression [27]. In support of this view, the loss of *kis* function causes homeotic transformations due to the failure to maintain transcription of *Hox* genes [28]. *kis* encodes a large protein (KIS-L) which is highly related to human CHD7 and other members of the chromodomain-helicase-DNA binding (CHD) subfamily of ATP-dependent chromatin-remodeling factors [28],[29]. KIS-L is associated with the vast majority of transcriptionally active regions of salivary gland polytene chromosomes, suggesting that it plays a relatively global role in transcription by Pol II [30]. KIS-L is not required for early stages of the transcription cycle, including the recruitment of Pol II to promoters and promoter clearance [30]. By contrast, the loss of *kis* function leads to a dramatic reduction in the levels of elongating Pol II and the elongation factors SPT6 and CHD1 associated with polytene chromosomes [30]. These findings suggest that KIS-L activates transcription by promoting an early stage of transcription elongation.

In addition to highly conserved ATPase domains, KIS-L and other CHD proteins contain chromodomains: a short domain implicated in the binding of methylated histone tails [31]. The presence of two chromodomains in KIS-L suggests that its ability to remodel chromatin and stimulate transcription may be regulated by the site-specific methylation of nucleosomal histones. Perhaps the best candidate for a chromatin modification that regulates KIS-L function is H3K4 di- or tri-methylation. Numerous H3K4 methyltransferases have been identified, including yeast SET1, its relatives in *Drosophila* and mammals, and the trithorax group proteins ASH1 and TRX [13],[23]. H3K4 methylation is found near the promoters of many active genes and is thought to stimulate transcription by promoting the association of multiple regulatory proteins with chromatin [23]. For example, CHD1 - a CHD protein related to KIS-L - directly binds methylated H3K4 (H3K4me) via its chromodomains [32],[33],[34]. Based on these findings, we suspected that the methylation of H3K4 by ASH1 and TRX might activate transcription by targeting KIS-L to promoters.

In this study, we sought to clarify the role of KIS-L in transcriptional regulation in vivo. By analyzing defects resulting from the loss of *kis* function, we found that KIS-L acts downstream of positive transcription elongation factor b (P-TEFb) recruitment to stimulate elongation by Pol II. KIS-L did not bind methylated histone tails in vitro or extensively co-localize with H3K4me on polytene chromosomes, as would be expected if this modification is necessary or sufficient for the recruitment of KIS-L to chromatin. Surprisingly, the loss of *kis* function led to a dramatic reduction in the levels of TRX and ASH1 associated with chromatin, accompanied by a significant increase in the level of H3K27 methylation. These findings suggest that KIS-L promotes transcription elongation and counteracts Polycomb group repression by recruiting the ASH1 and TRX histone methyltransferases to chromatin.

## Results

### KIS-L Acts Downstream of P-TEFb to Facilitate Elongation by Pol II

Previous studies in our laboratory suggested that KIS-L promotes a step in transcription downstream of the recruitment of Pol II to promoters [30]. The phosphorylation of the heptapeptide repeat Y_1_S_2_P_3_T_4_S_5_P_6_S_7_ of the C-terminal domain (CTD) of Pol II is highly regulated during the transcription cycle [4]. When recruited to promoters, the CTD is hypophosphorylated (Pol IIa). Phosphorylation of serine 5 of the CTD by the Cdk7 subunit of TFIIH is associated with promoter clearance and Pol IIo^ser5^ is enriched in promoter-proximal regions [4]. The subsequent phosphorylation of serine 2 of the CTD by the Cdk9 subunit of P-TEFb relieves pausing and stimulates the transition to active elongation [35]. Thus, Pol IIo^ser2^ is associated with the body of actively transcribed genes. Using antibodies sensitive to the phosphorylation state of the CTD, we previously demonstrated that the loss of *kis* function leads to a dramatic reduction in the level of Pol IIo^ser2^ - but not Pol IIa or Pol IIo^ser5^ - associated with salivary gland polytene chromosomes [30]. This observation suggested that KIS-L is required for a relatively early step in elongation by Pol II.

A key step in early elongation is the recruitment of P-TEFb to promoters [35]. P-TEFb (a heterodimer of CycT and Cdk9) has multiple functions: it phosphorylates serine 2 of the CTD; promotes the maturation of elongation complexes; counteracts promoter-proximal pausing induced by the DRB sensitivity-inducing factor (DSIF) and negative elongation factor (NELF); and stimulates the processivity of elongating Pol II [36]. The loss of P-TEFb function causes defects similar to those observed in *kis* mutants, including a reduction in the level of Pol IIo^ser2^, but not Pol IIa or Pol IIo^ser5^, associated with chromatin [37],[38]. We therefore suspected that KIS-L might be required for the recruitment of P-TEFb to promoters.

**Figure 1:**
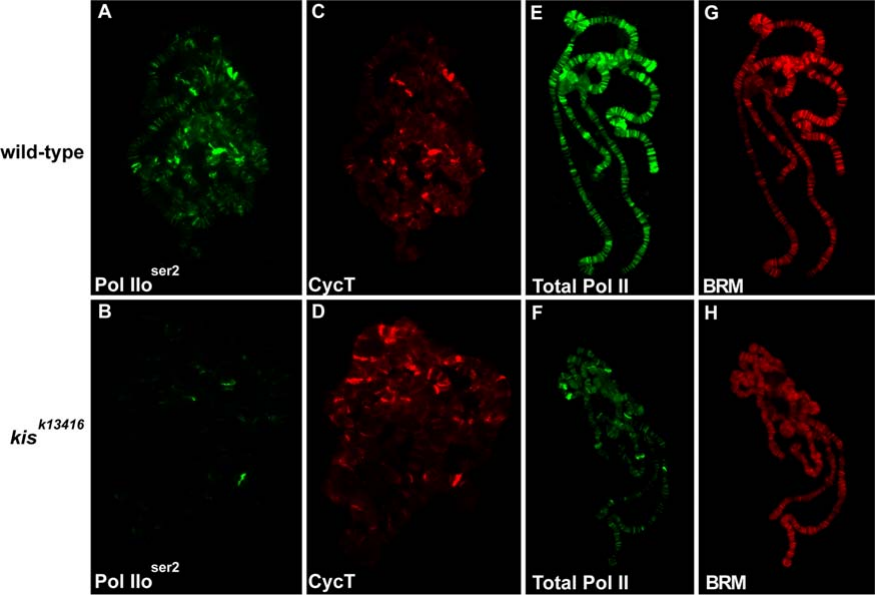
KIS-L facilitates an early stage in transcriptional elongation. Polytene chromosomes isolated from wild-type (A, C, E, and G) and *kis^k13416^* (B, D, F, and H) larvae were stained with antibodies against Pol IIo^ser2^ (A, B) and the CycT subunit of P-TEFb (C, D) or the CTD of RPB1 (that recognizes hypo- and hyper-phosphorylated forms of Pol II) (E, F) and BRM (G, H). The levels of total Pol II and Pol IIo^ser2^ are reduced in *kis* mutants while the levels of BRM and CycT are not altered.

To determine whether KIS-L is required for P-TEFb recruitment, we stained polytene chromosomes of wild-type and *kis^k13416^* larvae with an antibody against the CycT subunit of P-TEFb [39]. As expected, the chromosomal distribution of P-TEFb and Pol IIo^ser2^ overlap extensively in wild-type larvae (Figure 1A and C). Although the level of Pol IIo^ser2^ associated with polytene chromosomes is dramatically reduced in *kis^k13416^* larvae (Figure 1A and B), the loss of *kis* function has no obvious effect on either the level or distribution of CycT (Figure 1C and D). Thus, KIS-L affects a step in transcription downstream of the recruitment of P-TEFb to promoters.

How does KIS-L influence transcription? The reduced levels of Pol IIo^ser2^ on the salivary gland chromosomes of *kis* mutants could be due to either the decreased phosphorylation of serine 2 of the CTD or the stalling or loss of Pol II downstream of promoters. To distinguish between these possibilities, we stained polytene chromosomes of wild-type and *kis^k13416^* larvae with an antibody (CTD4H8) that recognizes hypo- and hyper-phosphorylated forms of Pol II [40]. The level of total Pol II associated with the salivary gland polytene chromosomes of *kis^k13416^* larvae was significantly reduced relative to wild-type (Figure 1E and F); by contrast, the level of chromatin-remodeling factor BRM was not altered (Figure 1G and H). These findings suggest that KIS-L prevents the stalling or loss of Pol II a relatively short distance downstream of promoters.

### KIS-L Co-localizes with the ASH1 and TRX Histone Methyltransferases on Polytene Chromosomes

We next investigated the functional relationship between KIS-L and the trithorax group proteins ASH1 and TRX. It has been reported that both ASH1 and TRX methylate H3K4 [18],[19],[20]; this covalent modification of chromatin is enriched near the promoters of many genes and is thought to recruit factors required for early events in the transcription cycle [23]. Like other CHD proteins, KIS-L has two adjacent chromodomains (CD1 and CD2) suggesting that it might directly interact with methylated histone tails. We therefore suspected that ASH1 and TRX might activate transcription by recruiting KIS-L to promoters.

**Figure 2:**
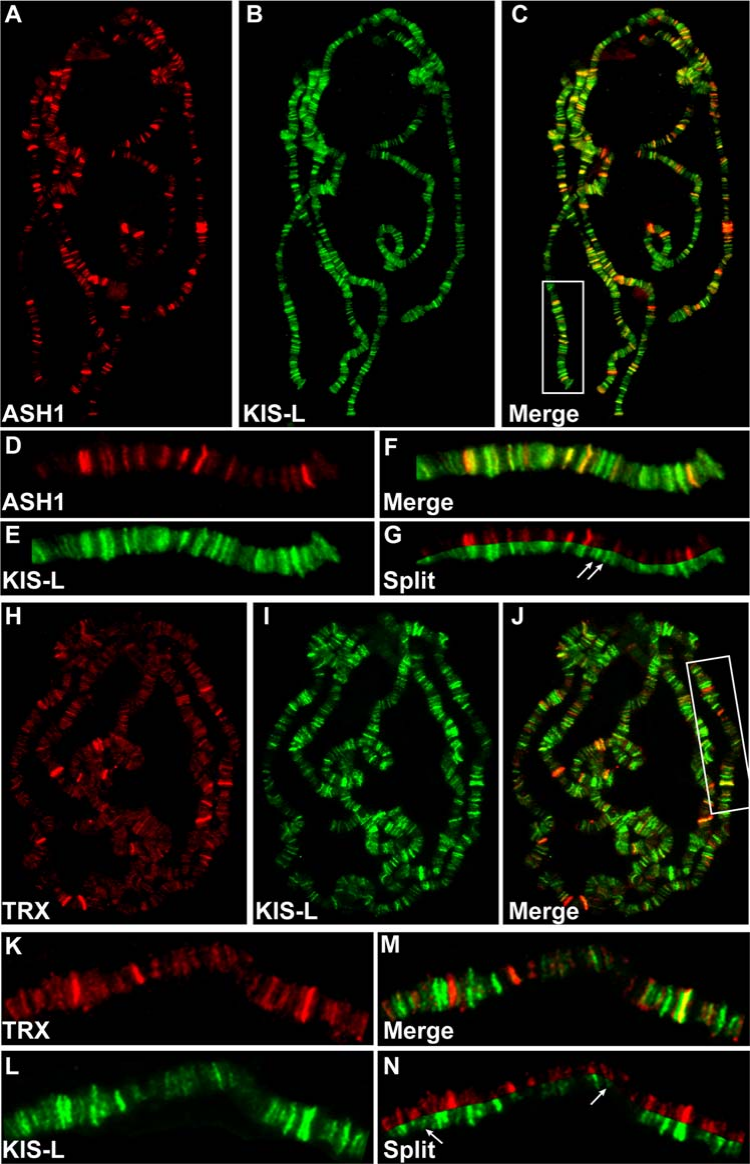
KIS-L co-localizes with the trithorax group proteins ASH1 and TRX on polytene chromosomes. A–C) The distributions of ASH1 (A, red) and KIS-L (B, green) on wild-type polytene chromosomes are shown together with the merged image (C). D–G: Magnification of the chromosome arm bounded by the white rectangle in C is shown. Arrows in G mark examples of KIS-L bands that do not overlap with ASH1. H–J) The distributions of TRX (H, red), KIS-L (I, green) and the merged image (J) are shown. K–N: represent the magnification of the chromosome arm bound by the white rectangle in J. The arrows in N represent bands of KIS-L that do not overlap with TRX.

As a first step toward testing this hypothesis, we compared the distributions of KIS-L, ASH1 and TRX on polytene chromosomes. As reported previously [41], ASH1 binds to approximately one hundred sites on salivary gland polytene chromosomes (Figure 2A). Strong TRX staining is observed at about 20 sites on polytene chromosomes [42],[43], but weaker signals are evident at many other sites (Figure 2H) [44]. The chromosomal distributions of KIS-L and ASH1 are strikingly similar, with overlapping signals observed at more than 95% of the binding sites of both proteins (Figure 2A–G). The chromosomal distributions of KIS-L and TRX are also similar, but not identical, with overlapping signals present at approximately 85% of the bindings sites of both proteins (Figure 2H–N). The co-localization of KIS-L, ASH1 and TRX at the majority of active genes is consistent with a close functional relationship between the three proteins.

### The Chromodomains of KIS-L Do Not Bind Methylated Histone Peptides in Vitro

**Figure 3:**
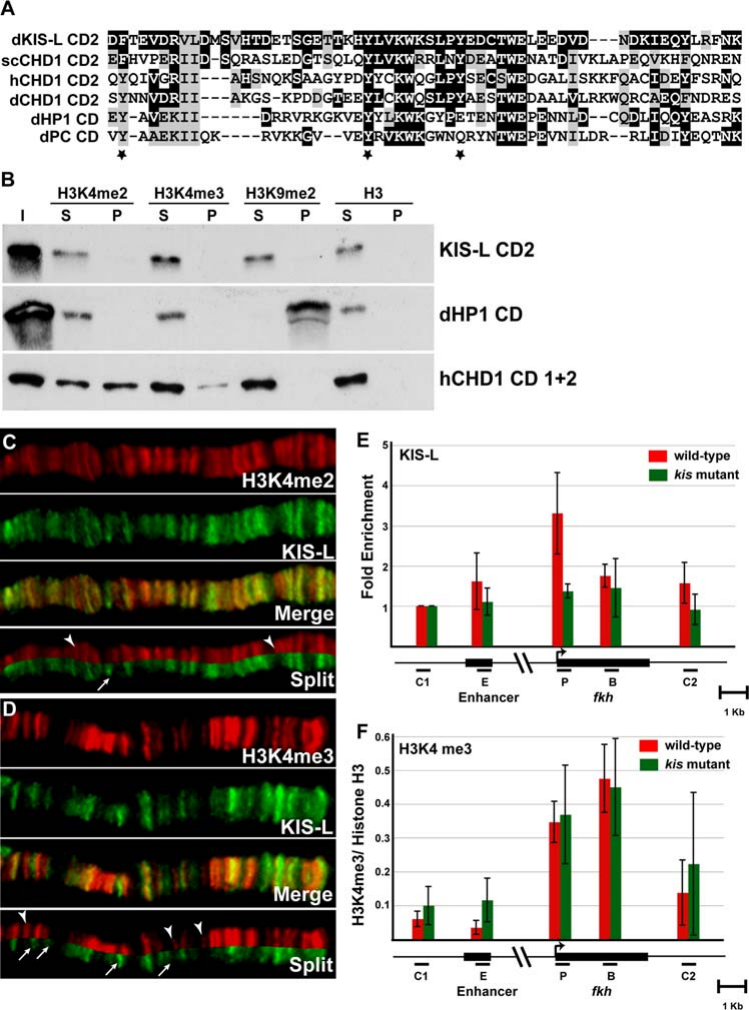
H3K4 methylation is not sufficient for the recruitment of KIS-L to chromatin. A) CD2 of KIS-L is aligned with CD2 of other CHD proteins and the chromodomains of *Drosophila* HP1 and PC. Identical and conserved amino acids are highlighted in black and grey, respectively. Aromatic amino acids that are important for binding of methylated histone tails by the CD2 of yeast CHD1 are marked by stars. B) The in vitro binding of HIS-tagged CD2 of KIS-L, CD1 and 2 of human CHD1 and the *Drosophila* HP1 chromodomain to histone H3K4me2, H3K4me3, H3K9me2 and histone H3 peptides were examined. Input (I), unbound protein (S) and the bound proteins (P) were detected by western blotting using anti-HIS tag antibody. Note that the chromodomains of HP1 and human CHD1, but not KIS-L, specifically bound methylated H3K9 and H3K4 peptides, respectively. C–D) The distributions of H3K4me2 (C, red) and H3K4me3 (D, red) were compared to that of KIS-L (C and D, green) on a representative region of wild-type polytene chromosomes. The arrowheads represent H3K4me2 and H3K4me3 bands that do not overlap with KIS-L, respectively, while the arrows represent bands of KIS-L that do not overlap with H3K4me2 and H3K4me3 bands, respectively. E–F) The distributions of KIS-L and H3K4me3 over the *fkh* gene were determined by ChIP using chromatin isolated from the salivary glands of wild-type (red bars) or *kis^k13416^* (green bars) larvae. A map of the *fkh* gene is shown below the X axis; black bars represent the primers used to amplify the following regions: C1: region upstream of *fkh*, E: *fkh* enhancer, P: *fkh* transcription start site, B: *fkh* body, C2: region downstream of *fkh*. For KIS-L, the percentages of DNA immunoprecipitated for regions E, P, B and C2 were normalized to the percentage of DNA immunoprecipitated for region C1 (E). The ratio of DNA immunoprecipitated with antibodies against H3K4me3 and histone H3 are shown for each region (F). Note that KIS-L is enriched over the transcription start site of *fkh* while H3K4me3 is enriched over both the transcription start site and the body of *fkh* gene. The bars represent the average of independent biological experiments (n = 4 for H3K4me3 and n = 5 for KIS-L) with the corresponding standard deviations.

CD2 of KIS-L is highly related to chromodomains that directly bind methylated histone tails (Figure 3A), including CD2 of yeast CHD1, which binds both di- and tri-methylated H3K4 [33]. This similarity suggested that KIS-L might directly bind methylated H3K4. To investigate this possibility, we examined the ability of recombinant KIS-L proteins to bind immobilized synthetic peptides corresponding to N-terminal histone tails. A recombinant protein corresponding to KIS-L CD2 (residues 1937 to 1997) did not bind unmodified histone H3 tails or a variety of methylated H3 tails (including H3K4me2, H3K4me3, and H3K9me2), even at relatively low (150 mM) salt concentrations (Figure 3B). By contrast, we were able to detect the binding of the *Drosophila* HP1 chromodomain to H3K9me2 using this assay (Figure 3B), as previously observed by others [45],[46]. Recent studies of the human CHD1 protein have shown that both CD1 and CD2 are required for binding of methylated H3K4 in vitro [32],[34]. While we were able to reproduce this result (Figure 3B), a comparable recombinant protein spanning CD1 and CD2 of KIS-L – as well as full-length KIS-L proteins from embryo extracts – bound both unmodified and methylated H3 and H4 tails (data not shown), presumably due to non-specific ionic interactions with the positively charged tails. We were therefore unable to determine if regions outside of CD2 enable KIS-L to bind methylated histone tails. Thus, although KIS-L CD2 failed to interact with methylated histone tails in vitro, it remains possible that the full-length KIS-L protein recognizes one or more histone modifications in vivo.

### H3K4 Methylation Is Not the Primary Determinant for the Recruitment of KIS-L to Chromatin

As an alternative approach for studying potential interactions between KIS-L and methylated histone tails, we compared the distributions of KIS-L and both di- and tri-methylated H3K4 on salivary gland polytene chromosomes. As expected for modifications associated with transcriptionally active regions, there is a high degree of overlap between KIS-L and both H3K4me2 and H3K4me3 (Figure 3C–D and Figure S1). However, there are many sites where KIS-L and these methyl marks do not overlap as well as considerable differences in the relative levels of KIS-L and H3K4 methylation at many sites (Figure 3C–D and Figure S1). These observations suggest that H3K4 methylation is not sufficient to recruit KIS-L to chromatin.

We next examined the relative distributions of H3K4 methylation and KIS-L at higher resolution using chromatin immunoprecipitation (ChIP) assays. We chose the *forkhead* (*fkh*) gene for these studies for several reasons. First, *fkh* is a relatively simple gene that is expressed in the salivary gland at high levels. A single enhancer located 9 kb upstream of the transcription start site activates *fkh* expression in this tissue [47]. Furthermore, TRX has been implicated in *fkh* expression [43] and we have demonstrated that KIS-L is associated with *fkh* by immuno-FISH (data not shown).

We examined the binding of KIS-L to the *fkh* gene by ChIP using chromatin isolated from the salivary glands of wild-type third instar larvae. Consistent with a role in early elongation, KIS-L is associated with the transcriptional start site of the *fkh* gene (Figure 3E). The enrichment of KIS-L with the transcriptional start site is about 3-fold over a control region upstream of *fkh* (Figure 3E, primer P vs. primer C1). This binding is reduced to background levels in chromatin isolated from *kis* mutant larvae, suggesting that the association of KIS-L with *fkh* is specific (Figure 3E). This finding is consistent with a recent study showing that KIS-L is associated with the *Ultrabithorax* (*Ubx*) promoter [25]. H3K4me3 is present at the transcription start site as well as the body of the *fkh* gene and does not precisely mirror the distribution of KIS-L (Figure 3F). These observations provide additional evidence that H3K4 methylation is not sufficient to recruit KIS-L to chromatin.

**Figure 4:**
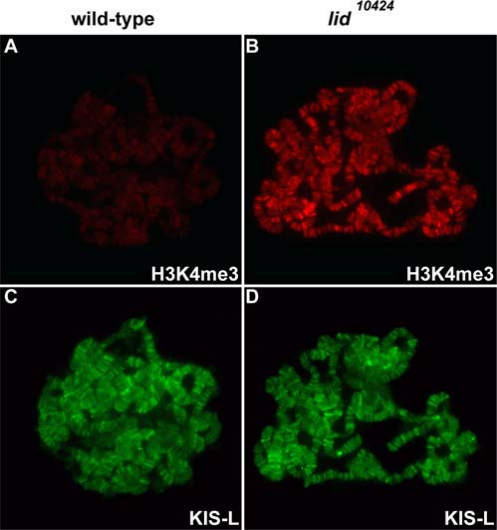
The association of KIS-L with chromatin is not altered in *lid* mutants. A–D) The levels of H3K4me3 (A, B, red) and KIS-L (C, D, green) on polytene chromosomes isolated from wild-type (A, C) and *lid^10424^* (B, D) larvae were examined by indirect immunofluorescence microscopy. Loss of *lid* function led to a dramatic increase in H3K4me3 without affecting the level of KIS-L associated with chromatin.

As an alternative approach for investigating the role of H3K4 methylation in KIS-L recruitment, we examined the effect of *little imaginal discs* (*lid*) mutations on the association of KIS-L with chromatin. *lid* encodes a H3K4me3 demethylase [48], [49], [50], [51]; larvae homozygous for the hypomorphic *lid^10424^* allele survive until the third larval instar and display elevated levels of H3K4me3 on their polytene chromosomes [50]. Increased trimethylation of H3K4 resulting from the loss of *lid* function has no obvious effect on the level of KIS-L associated with polytene chromosomes (Figure 4), suggesting that this covalent modification of chromatin does not mediate interactions between KIS-L and chromatin in vivo.

### Neither ASH1 nor TRX Is Required for the Association of KIS-L with Chromatin

**Figure 5:**
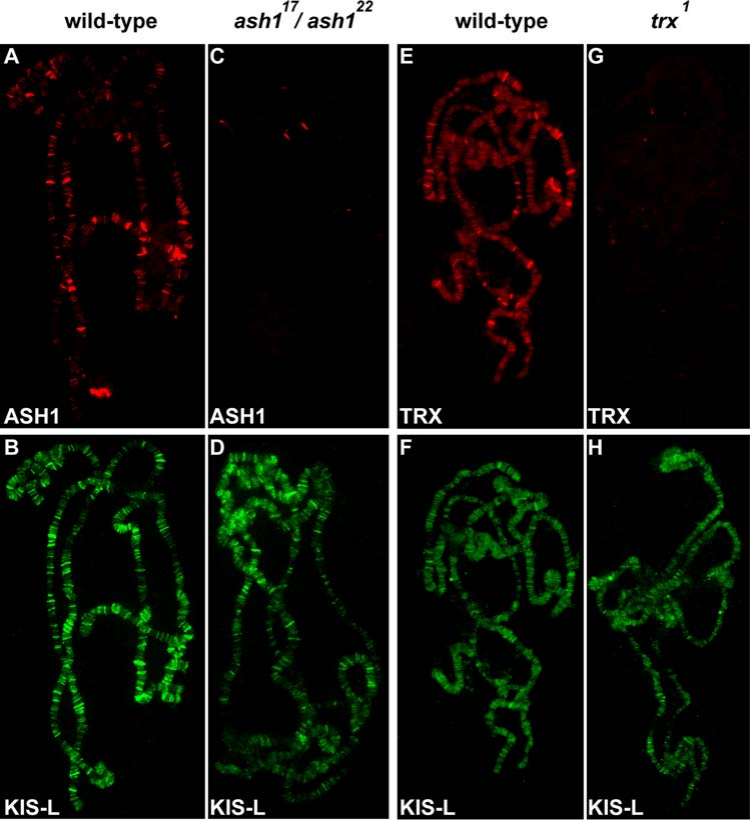
The association of KIS-L with chromatin is not altered in *ash1* and *trx* mutants. A–D) The association of ASH1 (A, C, red) and KIS-L (B, D, green) on salivary gland polytene chromosomes of wild-type (A, B) and *ash1^22^/ash1^17^* (C, D) larvae were detected by indirect immunofluorescence microscopy. E–H) The association of TRX (E, G, red) and KIS-L (F, H, green) on polytene chromosomes isolated from wild-type (E, F) and *trx^1^* (G, H) larvae were detected by indirect immunofluorescence microscopy. Neither ASH1 nor TRX is required for the binding of KIS-L to polytene chromosomes.

The above results led us to question our hypothesis that ASH1 and TRX recruit KIS-L to chromatin by methylating H3K4 in the vicinity of promoters. To clarify this issue, we examined whether the loss of ASH1 or TRX function alters the association of KIS-L with salivary gland polytene chromosomes. Individuals trans-heterozygous for the hypomorphic *ash1^22^* and *ash1^17^* alleles survive until the third larval instar and display significantly reduced levels of ASH1 on polytene chromosomes (Figure 5A and C) [41]. No obvious changes in the level or distribution of KIS-L were observed in these mutants relative to wild-type (Figure 5B and D), indicating that ASH1 is not required for the association of KIS-L with chromatin. Similar results were obtained using a conditional *trx* allele, *trx^1^*. At 29°C, *trx^1^* homozygotes survive until the third larval instar and display significantly reduced levels of TRX on polytene chromosomes (Figure 5E and G) [43]. We failed to detect obvious changes in the level or distribution of KIS-L on salivary gland chromosomes in *trx^1^* mutants (Figure 5F and H). Thus, neither the ASH1 nor TRX histone methyltransferases are required for the association of KIS-L with chromatin in vivo.

### KIS-L Is Required for the Association of ASH1 and TRX with Chromatin

**Figure 6:**
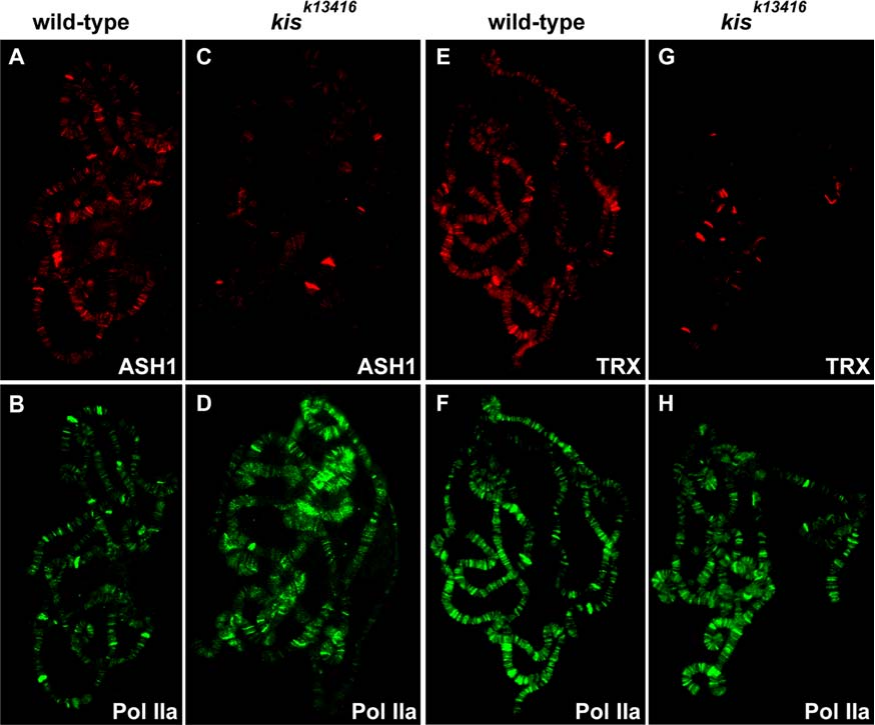
KIS-L is required for the association of ASH1 and TRX with chromatin. The distribution of ASH1 (A, C, red) and TRX (E, G, red) on salivary gland polytene chromosomes isolated from wild-type and *kis^k13416^* larvae were detected by indirect immunofluorescence microscopy. The chromosomes were also stained with an antibody against Pol IIa (B, D, F, H, green) as an internal control. The loss of KIS-L function dramatically reduces the levels of ASH1 and TRX, but not Pol IIa, associated with polytene chromosomes.

In some cases, chromatin-remodeling factors stimulate transcription by recruiting histone-modifying enzymes to promoters [52],[53]. We therefore examined if KIS-L is required for the association of ASH1 and TRX with chromatin. The loss of *kis* function resulted in a significant reduction in the levels of both ASH1 and TRX associated with polytene chromosomes (Figure 6A–G). This is unlikely to result from the decreased expression of ASH1 or TRX, as western blotting indicated that both proteins, though slightly reduced, were still present in *kis* mutants (Figure S2). A few residual bands of relatively strong ASH1 and TRX staining were observed in the mutants (Figure 6C and G), suggesting that the recruitment of the two trithorax group proteins to a small number of chromosomal sites may be independent of KIS-L. These results demonstrate that KIS-L is required for the recruitment of ASH1 and TRX to the majority of their target genes in vivo.

**Figure 7:**
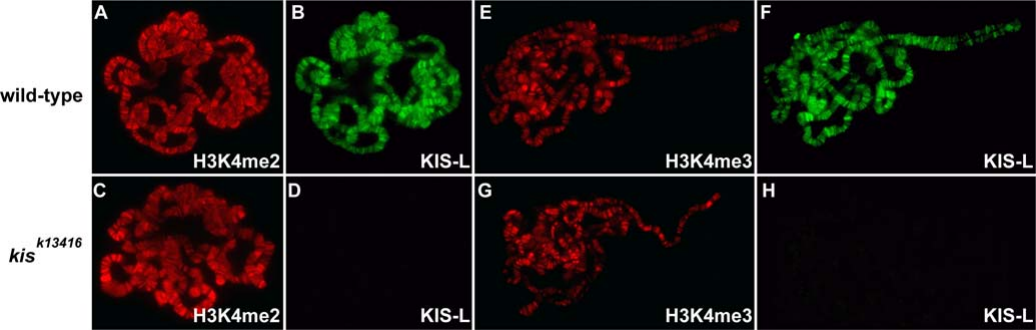
KIS-L is not a global regulator of H3K4 methylation. The distribution of H3K4me2 (A, C, red), KIS-L (B, D, F and H, green), and H3K4me3 (E, G, red) on salivary gland polytene chromosomes isolated from wild-type (A, B, E and F) and *kis^k13416^* (C, D, G and H) larvae were detected by indirect immunofluorescence microscopy. The loss of KIS-L function does not cause obvious changes in the overall level or distribution of either H3K4me2 or H3K4me3.

Although the substrate specificity of ASH1 is controversial, at least one previous study reported it to be responsible for the bulk of H3K4 methylation in the larval salivary gland [20]. This observation, together with our finding that KIS-L recruits ASH1 and TRX to actively transcribed genes, led us to investigate whether KIS-L is a global regulator of H3K4 methylation. Surprisingly, we did not observe a significant decline in either H3K4 di- or trimethylation on the polytene chromosomes of *kis^k13416^* mutant larvae, (Figure 7). Consistent with these data, the loss of KIS-L function had no effect on the level of H3K4 methylation over the promoter and body of the *fkh* gene, as assayed by ChIP (Figure 3F). We also examined the level of H3K4me2 and H3K4me3 on the salivary gland polytene chromosomes of *ash1* and *trx* mutant larvae. As observed in *kis* mutants, there was no significant decrease in H3K4 di- or trimethylation in either *ash1^22^*/*ash1^17^* or *trx^1^* larvae relative to wild type (Figure S3 and S4). These data strongly suggest that ASH1, TRX and KIS-L are not required for the bulk of H3K4 methylation in *Drosophila*.

### KIS-L Does Not Stably Interact with Other Trithorax Group Proteins in Vivo

Using gel filtration chromatography, we previously showed that the 574 kDa KIS-L protein is a subunit of large (∼1 MDa) complex, suggesting that it might also physically interact with other trithorax group proteins to regulate transcription by Pol II [30]. Gel filtration chromatography is sensitive to protein conformation, however, and erroneously high estimates of the native molecular masses of CHD1 and several other chromatin-remodeling factors have been obtained using this technique [30],[54],[55]. We therefore re-examined whether KIS-L functions as a subunit of a large protein complex by sedimenting *Drosophila* embryo extracts through sucrose density gradients (Figure S5). In contrast to the results we obtained using gel filtration chromatography, we found that the native and predicted molecular masses of the KIS-L protein are virtually identical; the 574 kDa KIS-L protein co-sedimented with ISWI complexes, which have a native molecular mass of ∼0.5 MDa (Figure S5). These data suggest that KIS-L acts as a monomer to regulate transcription by Pol II.

We also employed a genetic approach to investigate whether KIS-L acts as a monomer. Mutations in a conserved lysine residue in the ATP-binding site of chromatin-remodeling factors eliminate their catalytic activity without interfering with their ability to interact with other proteins. For chromatin-remodeling factors that function as subunits of protein complexes (e.g. BRM and ISWI), such catalytically inactive proteins exert strong, dominant-negative effects when expressed at high levels in vivo [56]. Unlike catalytically inactive forms of BRM and ISWI (ISWI^K159R^ and BRM^K804R^), the expression of high levels of an equivalent form of KIS-L (KIS-L^K2060R^) had no effect on the viability or development of a wide variety of tissues (data not shown). These findings provide additional, albeit indirect, evidence that KIS-L does not stably interact with other trithorax group proteins as a subunit of a chromatin-remodeling complex.

### Loss of *kis* Function Does Not Alter the Level or Distribution of Polycomb Group Proteins on Polytene Chromosomes

**Figure 8:**
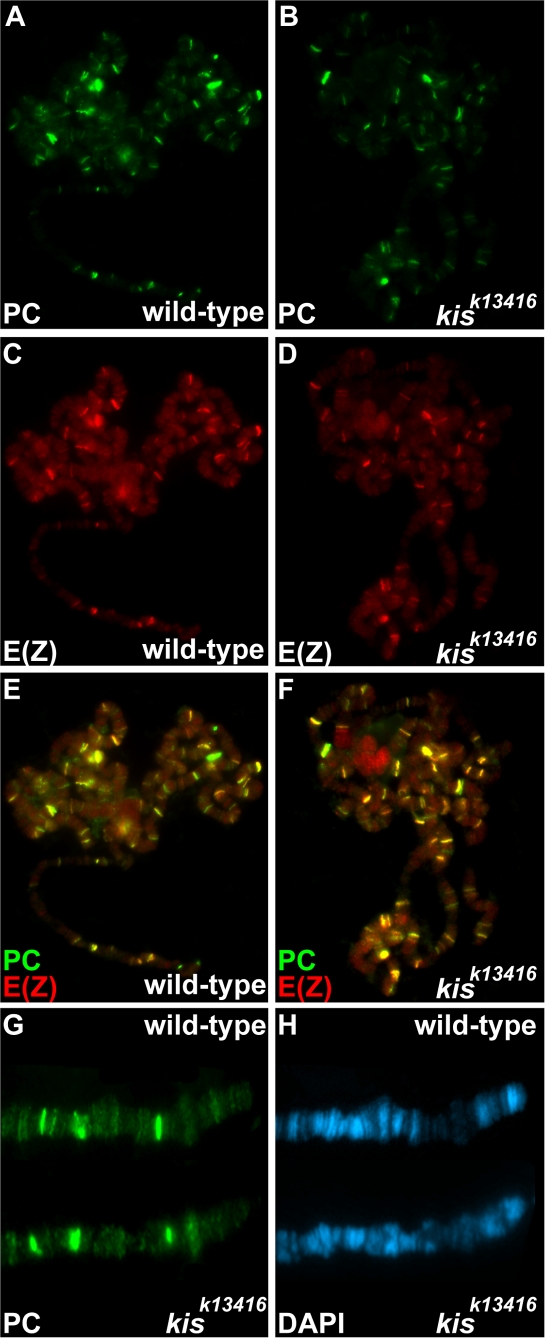
Loss of *kis* function does not alter the distribution or level of Polycomb group proteins. A–F) The distribution of PC (A, B, green) and E(Z) (C, D, red) and the merged images of PC and E(Z) (E, F) on polytene chromosomes isolated from wild-type (A, C, E) and *kis^k13416^* (B, D, F) larvae are shown. G–H) Comparison of the distribution of PC on the distal tip of the X chromosome of wild-type and *kis^k13416^* larvae (G), together with the corresponding DAPI staining (H). Note that the loss of *kis* function does not lead to obvious changes in the level or distribution of PC or E(Z).

Genetic studies have suggested that KIS-L and other trithorax group proteins counteract Polycomb group repression [26],[27]. Two complexes of Polycomb group proteins have been identified: PRC1 and PRC2 [14]. The E(Z) subunit of PRC2 methylates lysine 27 of histone H3; this modification is thought to promote the association of PRC1 with chromatin, thereby leading to hereditable gene silencing [16],[17]. Does KIS-L prevent the binding of either PRC1 or PRC2 to chromatin? As reported previously, the level of the PC subunit of PRC1 associated with salivary gland polytene chromosomes is similar in wild-type and *kis^k13416^* mutant larvae (Figure 8A and B) [30]. Similar results were obtained when we compared the level of E(Z) on salivary gland chromosomes of wild-type and *kis^k13416^* mutant larvae (Figure 8C and D). The loss of KIS-L function did not alter the number or distribution of PC binding sites (Figure 8G and H), and extensive co-localization of PC and E(Z) was observed in both wild-type and *kis* mutant larvae (Figure 8E and F). Thus KIS-L does not appear to influence the association of either PRC1 or PRC2 with chromatin.

### KIS-L Counteracts H3 Lysine 27 Trimethylation in Vivo

**Figure 9:**
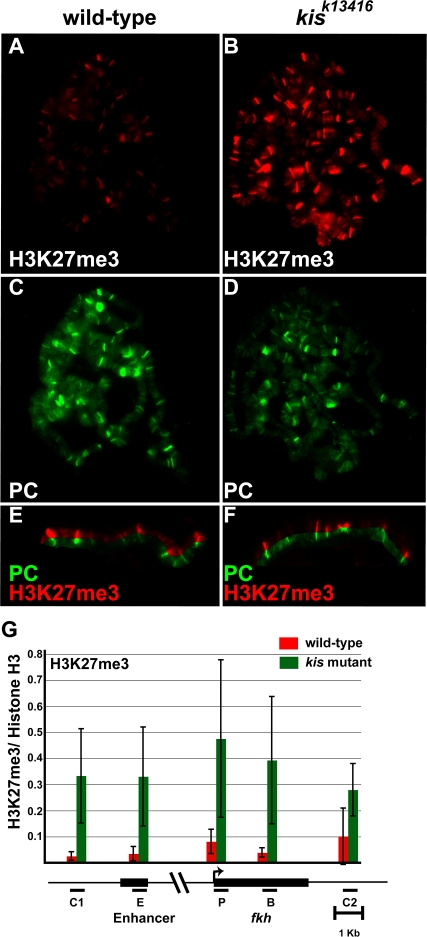
Mutations in *kis* lead to increased H3K27 methylation. A–D) The level and distribution of H3K27me3 (A, B, red) and PC (C, D, green) on salivary gland polytene chromosomes of wild-type (A, C) and *kis^k13416^* (B, D) larvae was examined by double-label indirect immunofluorescence microscopy. Split images of the distributions of H3K27me3 (red) and PC (green) on wild-type (E) and *kis^k13416^* (F) polytene chromosomes are shown. Loss of *kis* function results in an increase in the levels of H3K27me3 without altering the distribution of this modification on polytene chromosomes. For panels E and F, the levels of H3K27me3 were independently processed using Adobe Photoshop software to facilitate the comparison of the methyl mark and PC. G) The distribution of H3K27me3 over the *fkh* gene was determined by ChIP using chromatin isolated from the salivary glands of wild-type (red bars) or *kis^k13416^* (green bars) larvae. A map of the *fkh* gene is shown below the X axis; black bars represent the primers used to amplify the following regions: C1: region upstream of *fkh*, E: *fkh* enhancer, P: *fkh* transcription start site, B: *fkh* body, C2: region downstream of *fkh*. The ratio of DNA immunoprecipitated with antibodies against H3K27me3 and histone H3 are shown for each region. The bars represent the average of three independent biological experiments with the corresponding standard deviations. The loss of KIS-L function leads to a significant increase in H3K27me3 over the entire *fkh* region.

We previously noted that the majority (>80%) of PC binding sites in salivary gland polytene chromosomes are adjacent to sites of KIS-L binding [30]. The majority of sites of H3K27 methylation are also flanked on one or both sides by KIS-L (Figure S6). These observations, together with the lack of obvious changes in the level or distribution of PRC1 and PRC2 in *kis* mutants, suggested that KIS-L might counteract Polycomb group repression by antagonizing H3K27 methylation. To investigate this possibility, we stained salivary gland polytene chromosomes of wild-type and *kis^k13416^* mutant larvae with an antibody that specifically recognizes this histone modification. Loss of *kis* function results in a large (∼7 fold) increase in the level of H3K27me3 on polytene chromosomes (Figure 9A and B) without altering the level or distribution of PC (Figure 9C and D). A similar increase in H3K27 trimethylation was observed over the entire *fkh* gene of *kis^k13416^* mutant larvae by ChIP (Figure 9G).

E(Z) is responsible for the majority of H3K27 methylation in *Drosophila* [15], suggesting that the increased trimethylation of H3K27 in *kis* mutants is dependent on the action of Polycomb group proteins. Consistent with this view, the chromosomal distributions of PC and H3K27me3 are virtually identical (>90% overlap) in both wild type and *kis^k13416^* mutant larvae (Figure 9E and F). Although the level of H3K27 methylation is elevated in *kis^k13416^* mutants, the chromosomal distribution of both Polycomb group proteins and H3K27me3 appears to be relatively normal, suggesting that this increase is not due to the appearance of Polycomb group proteins or H3K27me at ectopic sites (Figure 9E and F). These findings suggest that that KIS-L antagonizes Polycomb group repression by counteracting H3K27 methylation catalyzed by the E(Z) subunit of PRC2.

**Figure 10:**
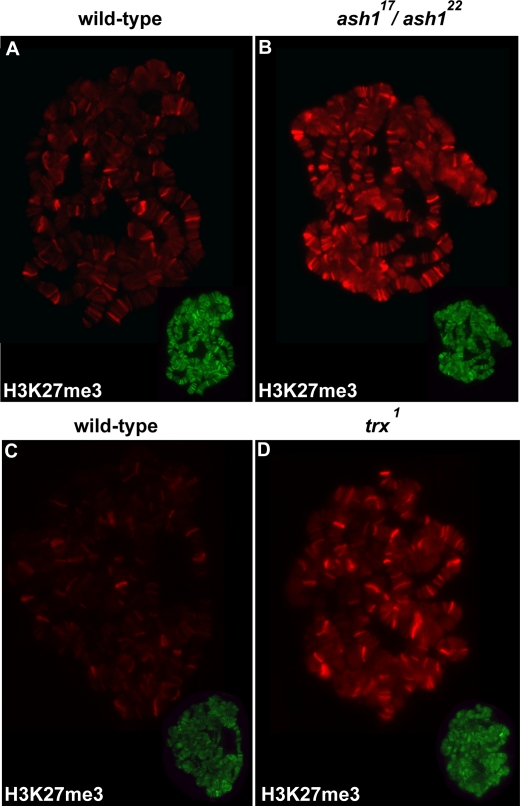
Loss of TRX and ASH1 function also leads to increased H3K27 methylation. The levels of H3K27me3 (A–D, red) on polytene chromosomes isolated from wild-type (A, C), *ash1^22^/ash1^17^* (B) and *trx^1^* (D) larvae were detected by double-label indirect immunofluorescence microscopy. H3K27me3 levels are higher on polytene chromosomes isolated from *ash1^22^/ash1^17^* and *trx^1^* mutants as compared to wild-type chromosomes. As an internal control, the chromosomes were simultaneously stained with antibodies against the RPB1 subunit of RNA Pol II (inset in lower right corner of A–D, green).

A recent study showed that loss of *ash1* function in the haltere discs of third instar larvae results in the spread of H3K27me3 into the coding region of the actively transcribed *Ubx* gene [25]. Thus, KIS-L may indirectly counteract H3K27 methylation by promoting the association of ASH1 with chromatin. To investigate this possibility, we compared the level and distribution of H3K27me3 on the salivary gland polytene chromosomes of wild-type and *ash1* mutant larvae. As observed in *kis* mutants, the level of H3K27me3 is dramatically elevated on the salivary gland polytene chromosomes of *ash1^22^*/*ash1^17^* larvae relative to wild-type (Figure 10A and B). A similar effect was observed in *trx^1^* homozygotes reared at 29° (Figure 10C and D). These findings suggest that KIS-L counteracts Polycomb group repression by promoting the association of ASH1 and TRX with chromatin.

## Discussion

### KIS-L Acts Downstream of P-TEFb to Promote Early Elongation by Pol II

The recent discovery that paused polymerases are present downstream of the promoters of many eukaryotic genes has stimulated great interest in the factors that regulate elongation by Pol II. Our findings suggest that KIS-L plays a critical role in this process. The initial stages of transcription, including the recruitment of Pol II to promoters and promoter clearance, are not affected by the loss of *kis* function. By contrast, the loss of *kis* function leads to a dramatic reduction in the level of elongating Pol II associated with chromatin. These findings suggest that KIS-L prevents the stalling or loss of Pol II a relatively short distance downstream of promoters.

How does KIS-L facilitate elongation? A key step in early elongation is the recruitment of P-TEFb to promoters. P-TEFb phosphorylates DSIF, NELF and the CTD of Pol II to counteract promoter-proximal pausing and stimulate elongation [36]. Treatment with flavopiridol, an inhibitor of P-TEFb kinase activity, causes transcription defects similar to those observed in *kis* mutant larvae [37],[38]. We therefore suspected that KIS-L might promote elongation by recruiting P-TEFb to promoters. The loss of *kis* function had no effect on the level of the CycT subunit of P-TEFb associated with chromatin, however, suggesting that KIS-L acts downstream of P-TEFb recruitment to stimulate elongation.

Nucleosomes present a physical barrier to Pol II that must be overcome to permit elongation; the failure to remove this barrier in *kis* mutants could cause the stalling or loss of Pol II downstream of promoters. Although the biochemical activities of KIS-L have not been characterized, all CHD proteins studied to date have ATP-dependent chromatin-remodeling activity in vitro [57]. It therefore seems likely that KIS-L facilitates elongation by altering the structure or spacing of nucleosomes in the vicinity of promoters. Precedent for this model is provided by work demonstrating that mammalian SWI/SNF stimulates elongation by remodeling nucleosomes downstream of the *Hsp70* promoter [58].

### Functional Interactions between KIS-L and Other Trithorax Group Proteins during the Transcription Cycle

A major goal of research on trithorax group proteins is to determine how they interact with each other and the general transcription machinery to activate gene expression and counteract Polycomb group repression. The majority of trithorax group proteins can be subdivided into three major classes based on their biochemical properties [26]. The first class consists of proteins involved in ATP-dependent chromatin remodeling, including KIS-L and the SWI/SNF subunits BRM, MOR, OSA and SNR1. The second class consists of histone-modifying enzymes, including ASH1 and TRX. The third class consists of mediator subunits, including Kohtalo and Skuld. Although BRM and KIS-L were both identified in genetic screens for extragenic suppressors of Polycomb mutations [27] and overlap extensively on polytene chromosomes [30], the two proteins appear to facilitate distinct steps in the transcription cycle. The BRM complex (like other SWI/SNF complexes) promotes the association of transcriptional activators with chromatin and is required for the recruitment of Pol II to promoters [59],[60], while KIS-L facilitates an early step in elongation by Pol II. Consistent with these observations, the loss of KIS-L function has no effect on the association of BRM with chromatin in vivo [30].

The presence of two chromodomains in KIS-L suggested that it might directly interact with nucleosomes in the vicinity of promoters that are methylated on H3K4 by ASH1 or TRX. However, several lines of evidence suggest that H3K4 methylation does not mediate interactions between KIS-L and chromatin. The chromodomains of KIS-L do not interact with H3K4 methylated peptides *in vitro* and we did not see a strong correlation between the distribution and level of KIS-L and methylated H3K4 on salivary gland polytene chromosomes of either wild-type or *lid* mutant larvae. Furthermore, neither ASH1 nor TRX are necessary for the association of KIS-L with chromatin in vivo. Although H3K4 methylation does not appear to play an important role in the recruitment of KIS-L to chromatin, it remains possible that this modification mediates transient interactions between KIS-L and its nucleosome substrate.

One of the more surprising outcomes of our work was the discovery that KIS-L is required for the association of ASH1 and TRX with many of their binding sites on polytene chromosomes. How does KIS-L promote the association of ASH1 and TRX with transcriptionally active genes? KIS-L might directly recruit the two proteins or indirectly promote their binding by influencing chromatin structure. Alternatively, KIS-L might promote the association of ASH1 and TRX with actively transcribed genes by promoting the transition from early to active elongation. A key step in this transition is the phosphorylation of serine 2 of the CTD repeat, which serves as a scaffold for the assembly of factors required for elongation and RNA processing [2]. The reduced levels of Pol IIo^ser2^ in *kis* mutants might therefore account for the decreased levels of ASH1 and TRX associated with chromatin.

Although we observed a dramatic decrease in the level of ASH1 and TRX associated with chromatin in *kis* mutants, we did not detect any obvious changes in the levels of BRM, Pol IIa, Pol IIo^ser5^ or P-TEFb. These observations strongly suggest that TRX and ASH1, like KIS-L, promote a stage of transcription downstream of initiation and promoter clearance. Our findings reinforce recent studies implicating ASH1 and TRX in transcription elongation in vivo. For example, TRX facilitates transcriptional elongation of heat shock genes [19] and the TRX-containing TAC1 complex is recruited downstream of the *Ubx* and *bxd* non-coding RNA promoters [44]. The levels of the 3′ end of *Ubx* mRNA and *bxd* non-coding RNA are reduced as compared to the 5′ end of these RNAs in *trx* mutants and the levels of SPT16, a member of the FACT elongation complex are also reduced in *trx* mutants [44]. These observations indicate that TRX might promote the processivity of the elongating polymerase by recruiting the FACT complex. MLL, the mouse homolog of TRX, is associated with the coding region of its target genes and the distribution of the elongating polymerase is altered in *Mll* mutants [61]. Although a role for ASH1 in elongation has not been firmly established, its binding downstream of the *Ubx* promoter in *Drosophila* and the human *poly(A) binding protein*, *cytoplasmic 1* gene in HeLa cells is consistent with a role in promoting transcription elongation [25],[62].

Does KIS-L promote elongation by recruiting ASH1 or TRX to promoters? Arguing against this possibility, we were unable to detect any significant alterations in the levels of Pol II on polytene chromosomes of *ash1* and *trx* single mutants (Figure 10 and data not shown). The failure to observe transcription defects in *ash1* and *trx* single mutants could be due to functional redundancy between the two trithorax group genes. Alternatively, KIS-L may promote elongation via ASH1 and TRX-independent mechanisms. Further work will be required to clarify the relative roles of KIS-L, ASH1 and TRX in transcription by Pol II.

### KIS-L Is a Negative Regulator of H3K27 Methylation

Several lines of evidence suggest that ASH1 and TRX activate transcription by counteracting Polycomb group repression. For example, in the absence of Polycomb group function, neither ASH1 nor TRX are required for *Ubx* transcription [24]. Furthermore, the loss of *ash1* function in haltere discs leads to the spread of H3K27me3 into the body of the *Ubx* gene, which is normally transcribed in this tissue [25]. Thus, ASH1 and TRX function may counteract Polycomb group silencing by interfering with H3K27 methylation.

Our discovery that KIS-L promotes the association of ASH1 and TRX with chromatin led us to investigate whether KIS-L might also counteract Polycomb group silencing. Polycomb group proteins are associated with relatively specific regions of chromatin (Polycomb-response elements, or PREs) as revealed by genome-wide ChIP assays [63],[64]. By contrast, H3K27me3 is found over broad chromatin domains adjacent to PREs, encompassing both the regulatory and coding regions of transcriptionally silent genes [63]. The loss of *kis* function led to a significant increase in the levels of H3K27me3 on salivary gland polytene chromosomes, suggesting that KIS-L may prevent the spreading of H3K27 methylation in the vicinity of PREs. Our observations provide a molecular explanation for the functional antagonism between *kis* and Polycomb group genes.

How does KIS-L antagonize H3K27 methylation? The steady-state level of H3K27 methylation is determined by multiple factors, including the level and activity of the E(z) methyltransferase; the accessibility of its nucleosome substrate; the frequency of nucleosome eviction; the frequency of histone H3 exchange, and the level and activity of histone H3K27 demethylases. The loss of KIS-L does not alter the distribution or level of PRC1 or PRC2 on polytene chromosomes, suggesting that it acts downstream of the E(Z) methyltransferase to counteract H3K27 methylation. Several H3K27 demethylases have been identified, including JMJD3 and UTX [65]. *Drosophila* UTX co-localizes with elongating Pol II, suggesting that H3K27 demethylation may be directly coupled to transcription elongation [66]. In humans, both JMJD3 and UTX are associated with complexes containing MLL proteins, the human orthologues of *Drosophila* TRX [67],[68]. Thus, KIS-L may indirectly antagonize H3K27 methylation by promoting elongation or the association of TRX with chromatin.

It is also possible that KIS-L counteracts H3K27 methylation by promoting the replacement of histone H3 by the histone variant H3.3. H3.3 displays covalent modifications associated with actively transcribed genes, including elevated H3K4 methylation and low levels of H3K27 methylation [69]. Elevated histone H3 replacement has also been observed at the binding sites of Polycomb and trithorax group proteins in the vicinity of *Drosophila Hox* genes [70]. The disruption of histone H3 replacement in cis-regulatory regions could therefore contribute to the increased levels of H3K27 methylation observed in *kis*, *ash1* and *trx* mutants. The exchange of histone H3 and H3.3 is also elevated in regions transcribed by Pol II [71]. By blocking transcription elongation and the resulting exchange of histone H3, the loss of KIS-L function could lead to elevated levels of H3K27 methylation in the body of genes. This would provide a straightforward explanation for why many transcriptionally active genes are refractory to Polycomb group silencing during early development.

### Implications for Human Disease

KIS-L is a member of a large family of CHD proteins that are conserved from flies to humans. Heterozygosity for loss of function mutations in CHD7, a human counterpart of KIS-L, leads to CHARGE syndrome, a serious developmental disorder affecting approximately one in 8,500 births [72]. Our findings suggest that CHARGE syndrome results from defects in transcriptional elongation, possibly due to the diminished recruitment of MLL complexes and elevated H3K27 methylation. Our findings also suggest a possible role for CHD7 in cancer through the recruitment of MLL fusion proteins, which have been implicated in several forms of leukemia [73]. Finally, numerous recent studies have revealed that the methylation of H3K27 by Polycomb proteins is critical for the maintenance of stem cell pluripotency in mammals [74]. By counteracting H3K27 methylation, CHD7 may promote the differentiation of pluripotent stem cells into specialized cell types. Further work will be necessary to test these hypotheses and clarify the role of KIS-L and its human counterparts in transcription, differentiation and disease.

## Materials and Methods

### Drosophila Stocks

Flies were raised on cornmeal/molasses/yeast/agar medium containing Tegosept and propionic acid. Strains are described in FlyBase (http://www.flybase.org) unless otherwise indicated. *kis^k13416^* is a recessive loss of function allele; homozygotes survive until late larval or early pupal stages, but express undetectable levels of KIS-L in salivary gland nuclei [30]. Oregon R was used as the wild-type strain for all experiments.

### Immunostaining of Polytene Chromosomes

Indirect immunofluorescence microscopy was used to examine the distribution of proteins on salivary gland polytene chromosomes [30],[56]. Primary antibodies used included goat antibodies against PC and KIS-L (Santa Cruz Biotech); rat antibodies against KIS-L [30]; rabbit antibodies against ASH1 [41], BRM [75], CycT [39], E(Z) [76], ISWI [77], KIS-L [30], TRX [43], Histone H3, H3K4me2, H3K4me3 and H3K27me3 (Upstate Signaling); and mouse antibodies against Pol IIa (8WG16), PoI IIo^ser2^ (H5), RPB1 (CTD4H8) (Covance) and His epitope tag (Anaspec). Salivary gland polytene chromosomes from third instar larvae were fixed for 5 minutes in 45% acetic acid/1.85% formaldehyde and stained with antibodies against ASH1, TRX, RPB1, Pol IIa, Pol IIo^ser5^ and KIS-L. To stain polytene chromosomes with antibodies against KIS-L, CycT, PoI IIo^ser2^, E(Z), PC, H3K4me2, H3K4me3 and H3K27me3, glands were dissected in 0.7% NaCl and fixed in 6 mM MgCl_2_, 1% citric acid and 1% Triton X-100 for 2 minutes. Secondary antibodies were obtained from Jackson ImmunoResearch Laboratories (West Grove, PA). Samples were mounted in Vectashield containing DAPI (Vector Laboratories). Images were captured using a Zeiss Axioskop 2 plus microscope equipped with an Axioplan HRm camera and Axiovision 4 software (Carl Zeiss, Germany). Merged and split images were generated using Adobe Photoshop CS3 software as previously described [56].

The levels of proteins associated with wild-type and mutant polytene chromosomes were compared by processing, capturing and analyzing the samples at the same time under identical conditions as described in Srinivasan et al. (2005). To quantify the increase in H3K27me3 levels in *kis* mutants, polytene chromosomes from wild-type and mutant larvae stained with antibodies against H3K27me3 were photographed using exposure times that yielded images of comparable intensity. The fold increase in H3K27me3 was calculated as a ratio of the average exposure times for the wild-type and mutant samples.

### Protein Expression and Binding Assays

Standard techniques were used to analyze proteins by SDS-PAGE and Western blotting [78]. To produce recombinant chromodomains, DNA encoding KIS-L chromodomain 2 (amino acids 1937–1997) was amplified using the primers 5′-GGAATTCCATATGCAGGACTTTACTGAAGT-3′ and 5′-CGGGATCCGATTTTGTTAAAGCGCAGGTA-3′. DNA encoding the HP1 chromodomain (amino acids 22–75) was amplified using the primers 5′-GGAATTCCATATGGAGGAGTACGCCGTGGA-3′ and 5′-CGGGATCCCTTGCGGCTCGCCTCGTACTG-3′. The amplified sequences were cloned between the *Nde* I and *BamH* I sites of pET-16b (Novagen). A pET-16b construct encoding chromodomains 1 and 2 of human CHD1 (amino acids 268 to 443) was generously provided by Sepidah Khorasanizadeh [32]. Chromodomains were expressed as His-tagged proteins in BL21pLysS (Stratagene) and purified by Ni^2+^ affinity chromatography under native conditions using the manufacturer's protocol (Qiagen). The binding of purified chromodomains to biotinylated peptides corresponding to N-terminal histone tails (Upstate) immobilized on strepavidin agarose (Upstate) was assayed as described in Pray-Grant et al. (2005).

### Analysis of Protein Expression in Salivary Glands

Identical numbers of salivary glands were dissected from third instar wild-type or *kis^k13416^* larvae, transferred to an eppendorf tube containing ice-cold 0.7% NaCl, and pelleted by brief centrifugation. After removing the supernatant, the glands were flash frozen in liquid nitrogen. Following the addition of boiling SDS-PAGE loading buffer, proteins were extracted from the glands by grinding with a pestle and analyzed by SDS-PAGE and western blotting using antibodies against ASH1 and TRX.

### Fractionation of Protein Extracts on Sucrose Gradients

Proteins were extracted from 0–16 hour *Drosophila* embryos as described previously [30] and fractionated by centrifugation through a 10 ml 5–30% sucrose gradient (in 50 mM Hepes, pH 7.6, 500 mM NaCl, 0.55% Tween-20, 10% glycerol, 1 mM MgCl_2_, 1 mM EDTA) at 4°C for 20 hours at 35,000 rpm in an SW41 rotor (Beckman Instruments). 500 µl fractions were collected and analyzed by SDS-PAGE and western blotting using antibodies against BRM, KIS-L and ISWI.

### Chromatin Immunoprecipitation (ChIP) and Quantitative-PCR

Chromatin was isolated from salivary glands of wild-type and *kis^k13416^* larvae [79] and analyzed by ChIP [80] using quantitative PCR (see Text S1 for details).

## Supporting Information

Figure S1 Distribution of KIS-L and H3K4 methylation on polytene chromosomes. The distributions of H3K4me2 (B, red) and H3K4me3 (E, red) are compared to that of KIS-L (A and D, green) on wild-type salivary gland polytene chromosomes by double-label indirect immunofluorescence microscopy. Merged images are shown in C and F. KIS-L is present at many, but not all, sites of H3K4 methylation.
(3.3 MB TIF)

Figure S2 Loss of KIS-L function does not dramatically alter the level of TRX and ASH1 in larval salivary glands. Proteins extracted from equal numbers of salivary glands of wild-type and *kis^k13416^* mutant larvae were analyzed by SDS-PAGE and western blotting using antibodies against TRX and ASH1. The loss of KIS-L function leads to only a modest reduction in the levels of TRX and ASH1, even though the salivary glands of *kis^k13416^* larvae are significantly (greater than two fold) reduced in size relative to those of wild-type larvae.
(0.4 MB TIF)

Figure S3 Loss of ASH1 function does not dramatically alter H3K4 methylation in vivo. The distribution of H3K4me2 (A, C, red), KIS-L (B, D, F and H, green), and H3K4me3 (E, G, red) on salivary gland polytene chromosomes isolated from wild-type (A, B, E and F) and *ash1^22^/ash1^17^* (C, D, G and H) larvae were detected by indirect immunofluorescence microscopy. The loss of ASH1 function does not cause obvious changes in the overall level or distribution of either H3K4me2 or H3K4me3.
(4.2 MB TIF)

Figure S4 Loss of TRX function does not dramatically alter H3K4 methylation in vivo. The distribution of H3K4me2 (A, C, red), KIS-L (B, D, F and H, green), and H3K4me3 (E, G, red) on salivary gland polytene chromosomes isolated from wild-type (A, B, E and F) and *trx^1^* (C, D, G and H) larvae were detected by indirect immunofluorescence microscopy. The loss of TRX function does not cause obvious changes in the overall level or distribution of either H3K4me2 or H3K4me3.
(5.7 MB TIF)

Figure S5 KIS-L is not a subunit of a large protein complex. The native molecular mass of KIS-L was determined by fractionating whole embryo extracts by sedimentation through a sucrose density gradient. Fractions were analyzed by SDS-PAGE and western blotting using antibodies against KIS-L, BRM and ISWI. The denatured molecular masses of KIS-L, BRM and ISWI are shown in kDa. KIS-L has a native molecular weight of slightly more than 0.5 MDa based on its sedimentation relative to the 1 MDa BRM and 0.5 MDa ISWI complexes.
(5.0 MB TIF)

Figure S6 Colocalization of KIS-L and H3K27 methylation. A) The distributions of KIS-L (green) and H3K27me3 (red) on wild-type salivary gland polytene chromosomes were compared by double-label immunofluorescence microscopy (A). B–E) The distributions of H3K27me3 (B), KIS-L (C), merged (D) and split (E) images corresponding to chromosome arm bounded by the white rectangle are shown. Note that KIS-L flanks many sites of H3K27me3 staining on polytene chromosomes.
(2.6 MB TIF)

Text S1 Supplemental material.
(0.02 MB DOC)
