# Supplementary figures and images for: Drosophila Kismet Regulates Histone H3 Lysine 27 Methylation and Early Elongation by RNA Polymerase II
Source: PLoS Genet. 2008 Oct 10;4(10):e1000217. doi: 10.1371/journal.pgen.1000217 (PMC2563034; doi:10.1371/journal.pgen.1000217)

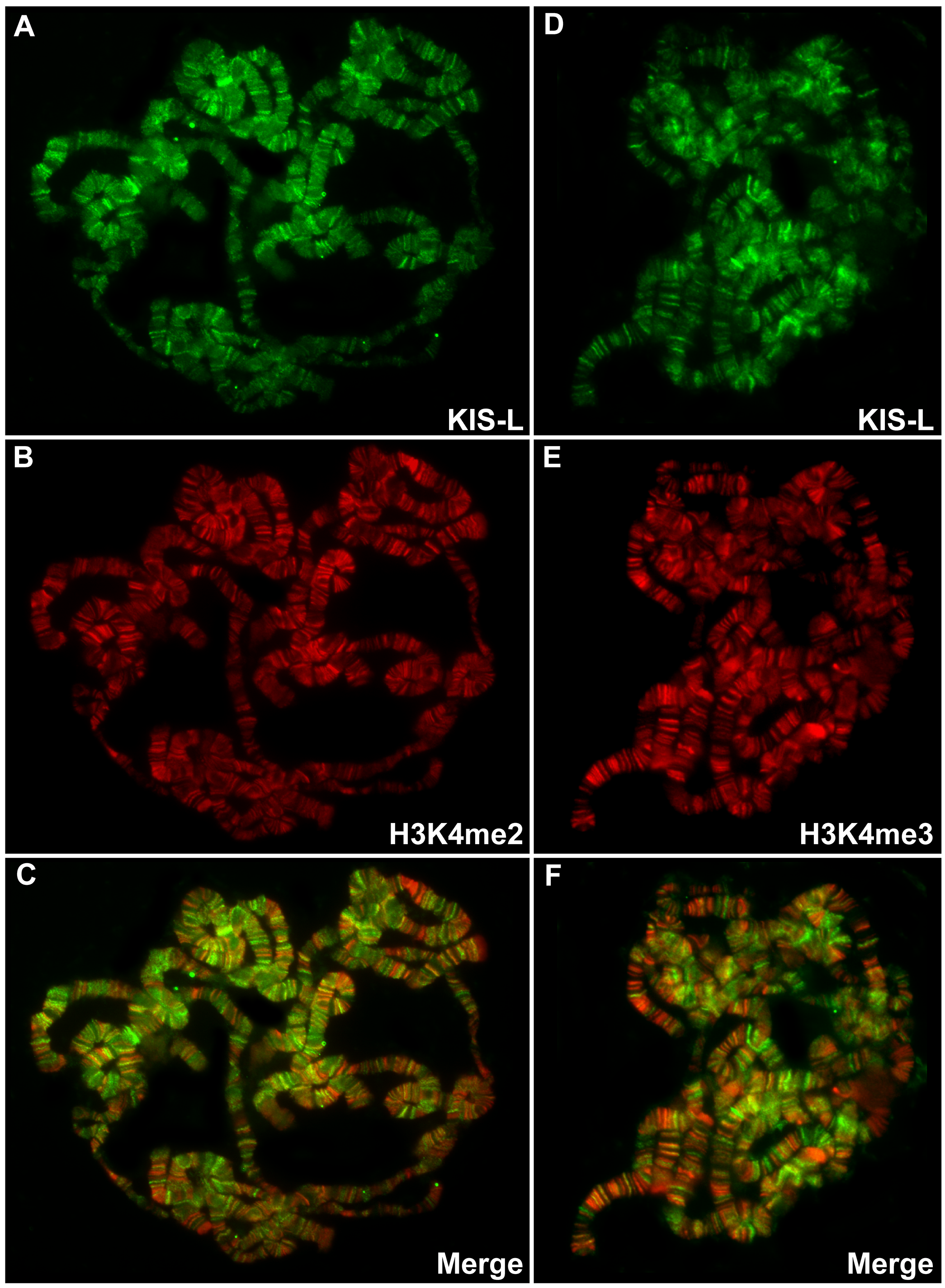

Supplement: Figure S1 — Distribution of KIS-L and H3K4 methylation on polytene chromosomes. The distributions of H3K4me2 (B, red) and H3K4me3 (E, red) are compared to that of KIS-L (A and D, green) on wild-type salivary gland polytene chromosomes by double-label indirect immunofluorescence microscopy. Merged images are shown in C and F. KIS-L is present at many, but not all, sites of H3K4 methylation. (3.3 MB TIF) [file pgen.1000217.s001.tif]

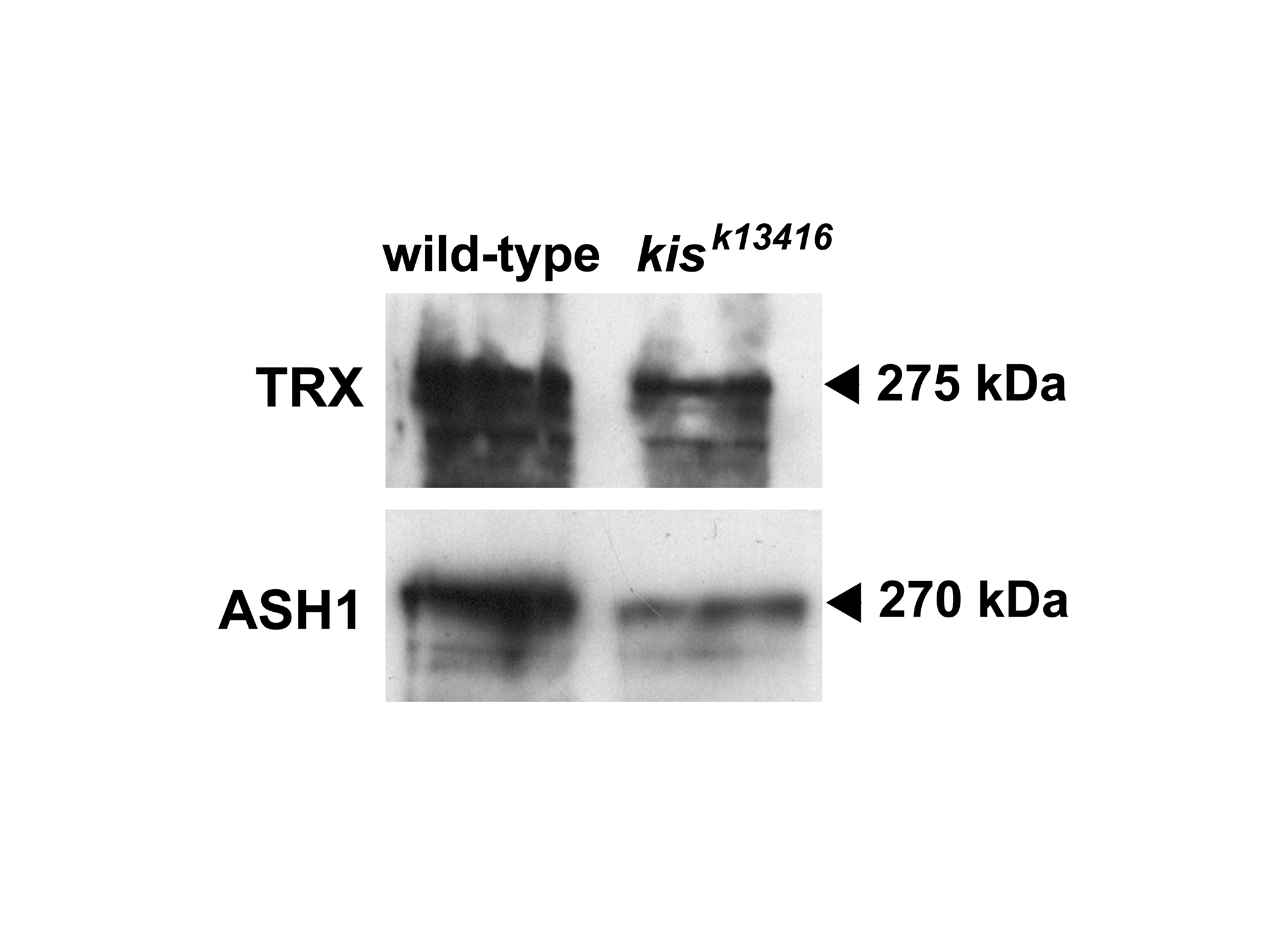

Supplement: Figure S2 — Loss of KIS-L function does not dramatically alter the level of TRX and ASH1 in larval salivary glands. Proteins extracted from equal numbers of salivary glands of wild-type and kisk13416 mutant larvae were analyzed by SDS-PAGE and western blotting using antibodies against TRX and ASH1. The loss of KIS-L function leads to only a modest reduction in the levels of TRX and ASH1, even though the salivary glands of kisk13416 larvae are significantly (greater than two fold) reduced in size relative to those of wild-type larvae. (0.4 MB TIF) [file pgen.1000217.s002.tif]

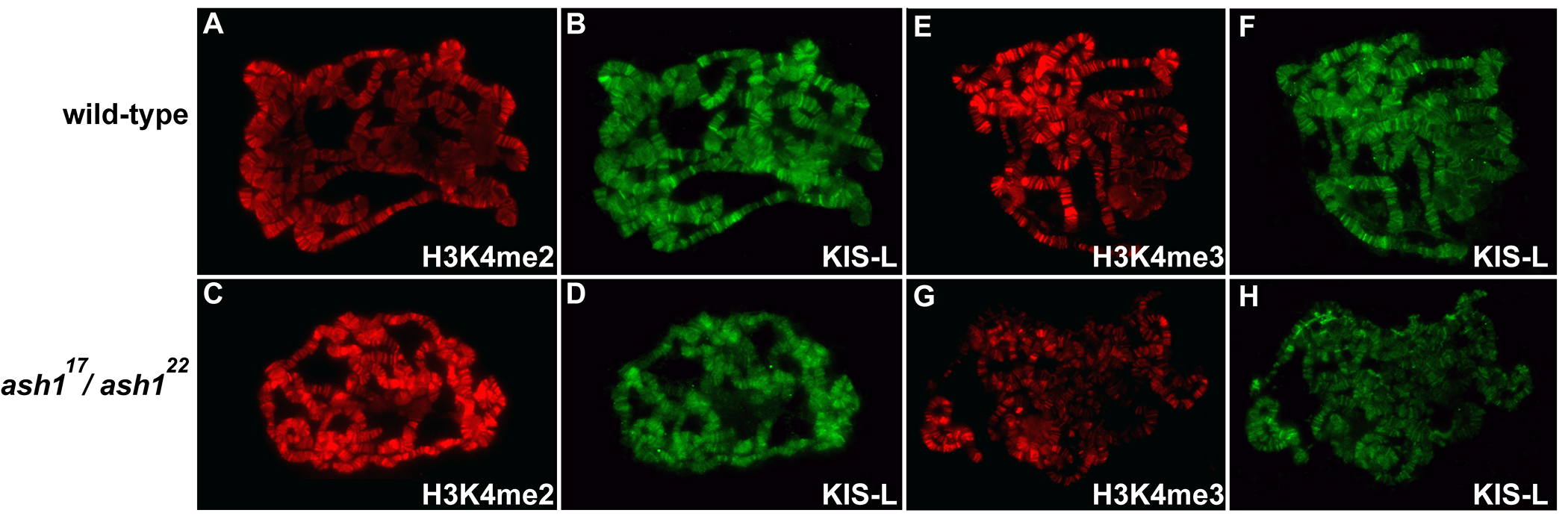

Supplement: Figure S3 — Loss of ASH1 function does not dramatically alter H3K4 methylation in vivo. The distribution of H3K4me2 (A, C, red), KIS-L (B, D, F and H, green), and H3K4me3 (E, G, red) on salivary gland polytene chromosomes isolated from wild-type (A, B, E and F) and ash122/ash117 (C, D, G and H) larvae were detected by indirect immunofluorescence microscopy. The loss of ASH1 function does not cause obvious changes in the overall level or distribution of either H3K4me2 or H3K4me3. (4.2 MB TIF) [file pgen.1000217.s003.tif]

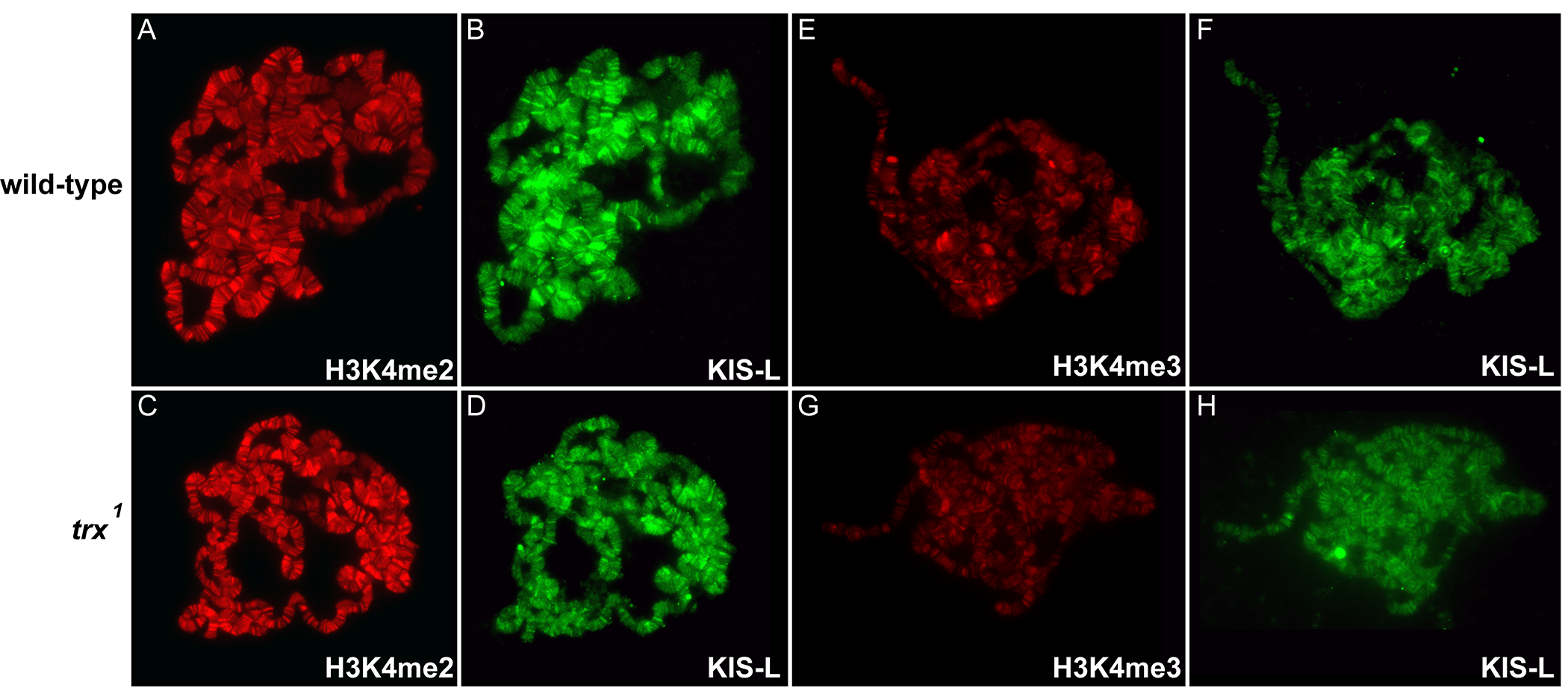

Supplement: Figure S4 — Loss of TRX function does not dramatically alter H3K4 methylation in vivo. The distribution of H3K4me2 (A, C, red), KIS-L (B, D, F and H, green), and H3K4me3 (E, G, red) on salivary gland polytene chromosomes isolated from wild-type (A, B, E and F) and trx1 (C, D, G and H) larvae were detected by indirect immunofluorescence microscopy. The loss of TRX function does not cause obvious changes in the overall level or distribution of either H3K4me2 or H3K4me3. (5.7 MB TIF) [file pgen.1000217.s004.tif]

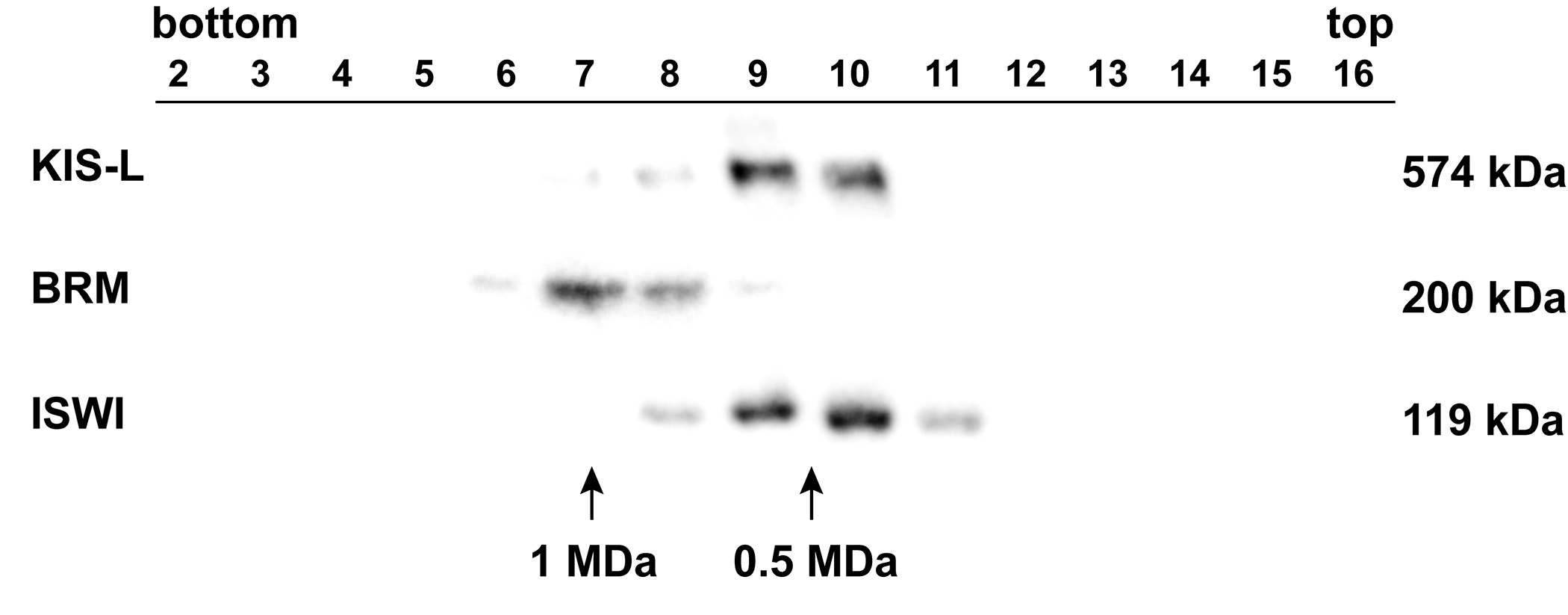

Supplement: Figure S5 — KIS-L is not a subunit of a large protein complex. The native molecular mass of KIS-L was determined by fractionating whole embryo extracts by sedimentation through a sucrose density gradient. Fractions were analyzed by SDS-PAGE and western blotting using antibodies against KIS-L, BRM and ISWI. The denatured molecular masses of KIS-L, BRM and ISWI are shown in kDa. KIS-L has a native molecular weight of slightly more than 0.5 MDa based on its sedimentation relative to the 1 MDa BRM and 0.5 MDa ISWI complexes. (5.0 MB TIF) [file pgen.1000217.s005.tif]

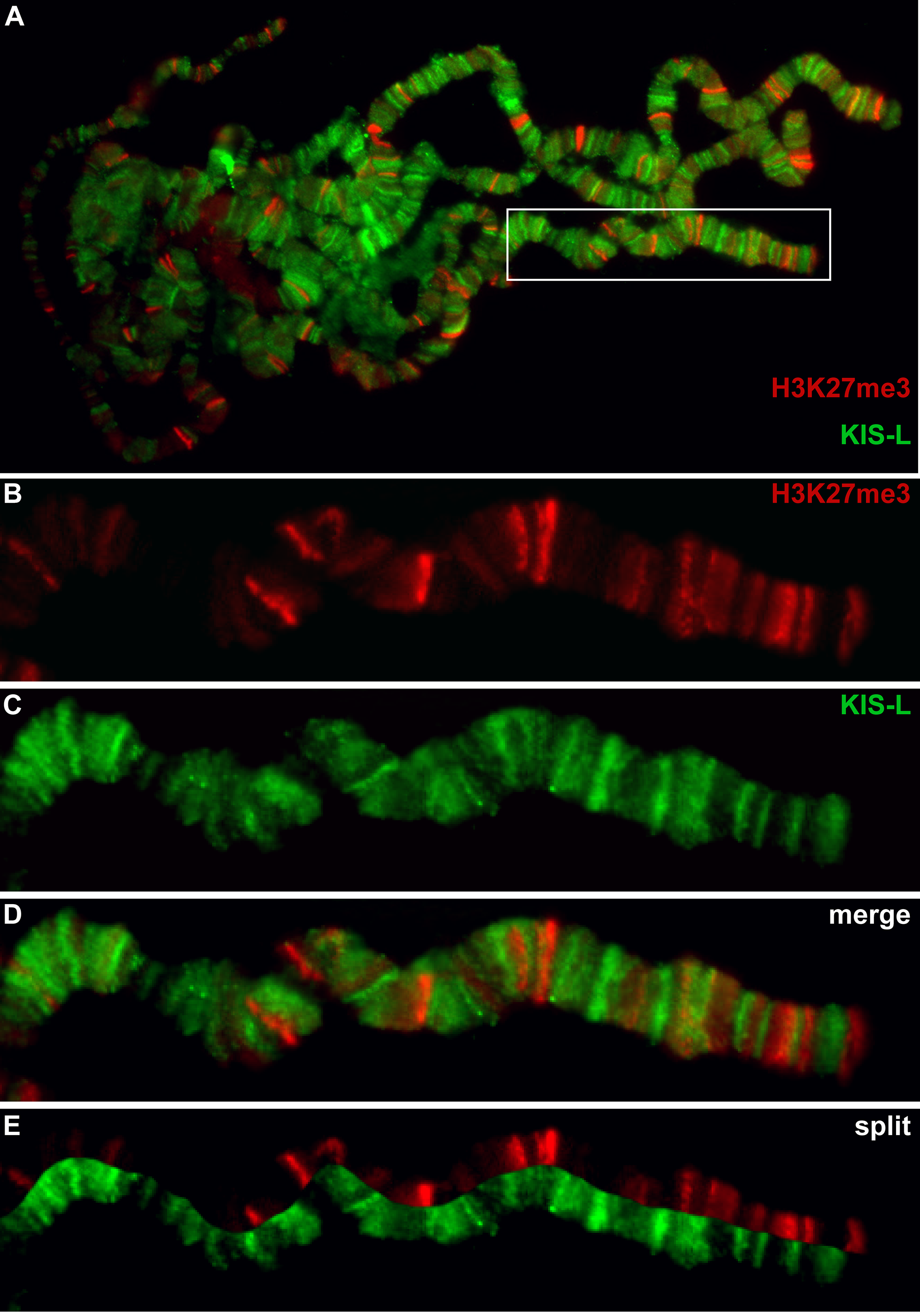

Supplement: Figure S6 — Colocalization of KIS-L and H3K27 methylation. A) The distributions of KIS-L (green) and H3K27me3 (red) on wild-type salivary gland polytene chromosomes were compared by double-label immunofluorescence microscopy (A). B–E) The distributions of H3K27me3 (B), KIS-L (C), merged (D) and split (E) images corresponding to chromosome arm bounded by the white rectangle are shown. Note that KIS-L flanks many sites of H3K27me3 staining on polytene chromosomes. (2.6 MB TIF) [file pgen.1000217.s006.tif]
