# Supplementary material for: Drosophila Kismet Regulates Histone H3 Lysine 27 Methylation and Early Elongation by RNA Polymerase II
Source: PLoS Genet. 2008 Oct 10;4(10):e1000217. doi: 10.1371/journal.pgen.1000217 (PMC2563034; doi:10.1371/journal.pgen.1000217)
Supplement: Text S1 — Supplemental material. (0.02 MB DOC) [file pgen.1000217.s007.doc]

**Supplemental Material**

**Materials and Methods**

*Chromatin immunoprecipitation*

50 pairs of salivary glands were isolated in ice-cold *Drosophila* Ringer’s solution (182 mM KCl, 46 mM NaCl, 3 mM CaCl_2_, 10 mM Tris-Cl, pH 7.2 with HCl). Dissected glands were transferred to Ringer’s solution and fixed for 10 minutes at room temperature with formaldehyde diluted in 20 mM triethanolamine, pH 7.0 to a final concentration of 1%. After washing with ice-cold TBS (140 mM NaCl, 20 mM Tris-Cl pH 7.5) followed by ChIP wash buffer (10 mM Tris-Cl pH 8.0, 1 mM EDTA, 0.5 mM EGTA, 0.5 mM PMSF), the fixed glands were frozen in liquid nitrogen and stored at -80°C.

Protein A sepharose (Bio-Rad) beads were washed with FA buffer (50 mM Hepes-KOH, pH 7.5, 1 mM EDTA, 1% Triton X-100, 0.1% sodium deoxycholate and 0.1% SDS) containing 150 mM NaCl and incubated with antibody overnight at 4°C. Fixed frozen glands were thawed on ice and FA buffer containing 275 mM NaCl, 1 mM PMSF and 1 mg/ml of complete protease inhibitor cocktail (Roche) was added. The glands were sonicated for 10 minutes (30 seconds of sonication with 30 seconds break) using a Diagenode Bioruptor (Molecular Diagnostics) to yield fragments ranging from 100 and 500 bp. Sonicated chromatin was diluted in FA buffer containing 275 mM NaCl to a final volume of 2 ml. 500 μl of this chromatin was saved as input and 500 μl was added to the protein A sepharose beads bound by antibody and rotated overnight at 4°C for immunoprecipitation. The beads were washed at room temperature with FA buffer containing 275 mM NaCl, FA buffer containing 500 mM NaCl, TLNEE (10 mM Tris-HCl, pH 8.0, 0.25 M LiCl, 1 mM EDTA, 0.5% NP-40, 0.5% sodium deoxycholate) and TE. Elution buffer (50 mM Tris-HCl pH 7.5, 10 mM EDTA and 1% SDS) was added to the beads and incubated at 70°C for 10 minutes. The beads were centrifuged at 10,000g for 2 minutes and the supernatant collected. The beads were washed with TE and the supernatant pooled with the previous supernatant. Proteinase K was added at a final concentration of 1 mg/ml to the pooled supernatants and the tubes were incubated at 45°C for 1 hour. The tubes were transferred to 70°C overnight to reverse the crosslinking. DNA was precipitated with ethanol after two rounds of phenol and chloroform extraction. The DNA was resuspended in 25 μl of TE.

*Quantitative-PCR*

Quantitative PCR was carried out using qPCR MasterMix Plus for SYBR® Green I Low ROX (Eurogentec) and Corbett Rotor Gene 6000 (Corbett Life Science) PCR machine. The PCR reactions were carried out in triplicate. The amount of immunoprecipitated DNA shown as a percentage of the input DNA was calculated as follows:

% IN = [(efficiency of primer)^-(Ct^_IP_^-Ct^_IN_)] x 100

Ct_IP_ = Threshold cycle for immunoprecipitated DNA, Ct_IN_ = Threshold cycle for input DNA.

The primers used to amplify specific DNA sequences included 5’ACCCGAGTCGAGTCACAGTGATTG3’ and 5’GTTTGCGTTTGGCATTTGTTGG3’ for the control region upstream of *fkh*; 5’GTCCTATACCGACCCATTCCCG3’ and 5’TAGGGAAGGTGCTCGAACTCGG3’ for the control region downstream of *fkh*; 5’CCAGTTCCCGAAAAACCATCTCATC3’ and 5’CACGGCGGTGGCACTCTATTAGC3’ for the *fkh* enhancer; 5’TCGAGCGGACCAGCAGCTAAAG3’ and 5’TGGGGATTTTTGTTGTCTGCCG3’ for the *fkh* transcription start site; and 5’CGAGAACGGGTGCTATTTGCGG3’ and 5’GCCTCCTTGTGGTGCTGATGATGG3’ for the body of the *fkh* gene.
